# Is industrial pollution detrimental to public health? Evidence from the world’s most industrialised countries

**DOI:** 10.1186/s12889-021-11217-6

**Published:** 2021-06-18

**Authors:** Mohammad Mafizur Rahman, Khosrul Alam, Eswaran Velayutham

**Affiliations:** 1 School of Business, University of Southern Queensland, Toowoomba, QLD 4350 Australia; 2 Department of Economics, Bangabandhu Sheikh Mujibur Rahman Science and Technology University, Gopalganj, 8100 Bangladesh

**Keywords:** Health status, Industrial pollution, Unbalanced panel data, Industrialised countries, Driscoll and Kraay’s standard error, C33, I10, O14, Q53

## Abstract

**Background:**

Industrial pollution is considered to be a detrimental factor for human health. This study, therefore, explores the link between health status and industrial pollution for the top 20 industrialised countries of the world.

**Methods:**

Crude death rate is used to represent health status and CO_2_ emissions from manufacturing industries and construction, and nitrous oxide emissions are considered to be indicators of industrial pollution. Using annual data of 60 years (1960–2019), an unbalanced panel data estimation method is followed where (Driscoll, J. C. et al. Rev Econ Stat, 80, 549–560, 1998) standard error technique is employed to deal with heteroscedasticity, autocorrelation and cross-sectional dependence problems.

**Results:**

The research findings indicate that industrial pollution arising from both variables has a detrimental impact on human health and significantly increases the death rate, while an increase in economic growth, number of physicians, urbanisation, sanitation facilities and schooling decreases the death rate.

**Conclusions:**

Therefore, minimisation of industrial pollution should be the topmost policy agenda in these countries. All the findings are consistent theoretically, and have empirical implications as well. The policy implication of this study is that the mitigation of industrial pollution, considering other pertinent factors, should be addressed appropriately by enunciating effective policies to reduce the human death rate and improve health status in the studied panel countries.

## Introduction

The link between environmental pollution and human health is discussed in a variety of literature, and is still a crucial issue of research, especially in the current COVID-19 pandemic situation. Environmental pollution contributes to climate change, which has various negative impacts on human health like perinatal disorders, infant mortality, respiratory disorders, allergies, malignancies, cardiovascular disorders, increase in oxidative stress, endothelial dysfunction, and mental disorders [1, 2]. The impacts on health can be so severe that they lead to death; nearly 7 million people die every year from the interaction of fine particles in polluted air (Goal- 3 of SDGs, [3]). Global environmental pollution is largely a result of people’s activities through urbanization, industrialization, large-scale petrochemical use, power generation, heavy industry, and mining and exploration, all of which adversely affect the health of local communities through their working and residential actions [4, 5]. Therefore, the matter of pollution distress has attracted contemporary global attention due to its acute long-run consequences on human health. Despite this global attention, there are still some policy uncertainties in the existing strategies due to the lack of a wide-ranging and comprehensive study that clearly addresses the adverse impact of industrial pollution on human health status.

Against this backdrop, this study has considered the connection between industrial pollution and health status in the world’s 20 most industrialised countries (see section 3.1). These countries have a total population of 4.57 billion (60% of the world’s population) where the total real GDP is US$66347.02 billion, reflecting 78.18% of the world’s real GDP [6]. The industrial value addition of these countries is US$17,783.95, which is 75.38% of world’s total industrial value addition [6]. The average crude death rate in this region is 8.23 per 1000 population, while the total CO_2_ emissions are 25,576.7 million tonnes covering 74.85% of the world’s emissions, and the nitrous oxide emissions are 18,37,026.82 thousand metric tons of CO_2_ equivalent, which is 58.25% of the world’s emissions [6, 7]. Hence our study is comprehensive and explores a vital issue in relation to both the environment and human health.

Some past studies (see [8–17], among others) have attempted to discover the factors that have impacts on human health and the death rate. However, based on their contradictory findings it has been difficult to draw conclusive and comprehensive guidelines for formulating certain policy initiatives. An inclusive and rigorous probe is necessary to achieve effective policy recommendations. From this perspective, this study is a conscientious venture to reduce the death rate by mitigating industrial pollution, considering the other controlled variables like economic growth, medical attention, drinking water services, sanitation services, secondary school enrolment and urbanisation in a panel of world’s most 20 industrialised countries.

The rationale for considering the desired variables lies in both the theoretical and conceptual notions and previous literary works. The CO_2_ and nitrous oxide emissions create industrial pollution which leads to the increase in the human death rate by generating pollution borne diseases ([12, 13, 17–23]; and [24]). Similarly, having access to the required number of physicians also plays an important role in reducing the mortality rate by providing more effective health care services ([11, 25–28]; and [29]). The access to clean water facilities, another vital element, decreases the death rate by satisfying basic needs (see [14, 16, 27, 30, 31]). Adequate and sufficient sanitation facilities also play a significant role in improving human health and reducing the mortality rate by maintaining safety and hygiene [8, 14–16, 27, 30–32]. In the same way, education facilities help to increase awareness about health consciousness and this may play a role in lowering the death rate ([10, 33–35]; Ray and Linden, 2020 [8, 25];). While a greater urbanisation rate increases different health related amenities which reduces mortality rate on the one hand, it also increases mortality by increasing pollution due to different urban activities ([9, 16, 17, 32, 36, 37]; and [27]). Therefore, more detailed investigation is still needed to assess the role of associated elements on human health.

The major contributions of this study can be noted as: (i) to the best of our knowledge, this is the first study in the literature that investigates the causative elements of the death rate in 20 of the world’s most industrialised countries; (ii) this study has used the most recent and wide-ranging data period of 60 years (1960-2019); (iii) the robust outcomes are achieved by employing an rigorous econometric approach like Driscoll and Kraay’s [38] standard error technique (details are in section 3.3); (iv) comprehensive policy recommendations, based on the results, are delivered for researchers and policy makers to address the issue of industrial pollution along with other relevant factors for reducing the death rate and improving human health by undertaking effective policy measures.

This research is designed in the following manner: following the introduction, section 2 provides the review of the past literatures; section 3 explains the data and methodology; section 4 displays and analyzes the empirical results; and section 5 provides the conclusion and policy recommendations.

## Literature review

Industrial pollution has many adverse consequences on human health and may be a cause of death because of respiratory, lung and cardio-related diseases (see [12, 13, 17–23]; and [24], among others). Leogrande et al. [13] discovered the significant impact of industrial air pollution on respiratory mortality in the Taranto area, Southern Italy by applying a difference-in-differences approach from the data of 1998–2014. Further, Karimi and Shokrinezhad [12] observed that PM_2.5_, PM_10_, CO, NO_2_, and SO_2_ were positively and significantly linked with both infant and child under-five mortality for 27 countries for the data period of 1992–2018. Bauleo et al. [19] found a positive association between industrial PM_10_ and mortality from non-accidental causes, all cancers, and cardiac diseases, for the industrial area of Civitavecchia, Italy. Similarly, Næss et al. [21] identified that the air pollutant indicators (NO_2_, PM_10_, and PM_2.5_) had a significant effect on all causes of death for both men and women in Oslo, Norway. Similar findings were also identified in the works of Anwar et al. [18] for 12 of the most vulnerable Asian countries, Shan et al. [23] for China, Lehtomäki et al. [20] for Nordic countries, Rajak and Chattopadhyay [22] for India, and Brito [39] for Portugal. Clancy et al. [40] maintained that control of the particulate air pollution significantly reduces the death rate from the comparison of pre and post 72 months ban on coal sales in Dublin, Ireland. In case of CO_2_ related pollution, Duong and Jayanthakumaran [41] found that an increase in CO_2_ emissions caused poor health for 60 provinces in Vietnam. Shobande [16] also demonstrated that the CO_2_ emissions increased infant and child mortality in the case of 23 African countries. Dedoussi et al. [42] and Im et al. [43] obtained evidence of pre-mature mortality due to cross state air pollution in the USA, and in four Nordic countries, respectively. In the case of the COVID-19 pandemic, Fareed et al. [24] ascertained that air pollution led to the increase of COVID-19 related mortality in Wuhan, China where daily data from 21 January 2020–31 March 2020 were used. Similar findings of COVID-19 related mortality were also observed by Coker et al. [44] for northern Italy, Isphording and Pestel [45] for Germany, Marquès et al. [46] for Spain, and Gupta et al. [47] for nine Asian cities. On the other hand, Karuppasamy et al. [48] found that the improved air quality due to reduced pollution decreased the mortality rate due to COVID-19 for India. However, Cheung et al. [49] obtained the statistically insignificant impact of the air pollution on cardio-respiratory mortality in Hong Kong. In their studies, the researchers did not focus on the rigorous estimation regarding the impact of the CO_2_ emissions and nitrous oxide emissions as outcomes of industrial pollution on the death rate, considering the panel of most industrialised countries.

Some works that considered CO_2_ emissions on the relationship with other variables were also found; in the literature (see Mehmood [50] for Singapore, and Mehmood and Tariq [51, 52], and Mehmood et al. [53, 54] for South Asian countries; Mehmood et al. [50] for 3 developing countries, and Mehmood [54] for South Asian countries). However, these studies did not show the impact of CO_2_ emissions, along with other relevant factors, on human health.

Thus, more critical analysis regarding the effect of industrial pollution on human health covering the most industrialised countries deserves further attention.

The death rate in a country is also related to the number of physicians employed in that country (see [11, 25–28]; and [29], among others). In this context, Jebeli et al. [11] found that there is a strong reverse correlation between the number of physicians and the crude death rate in the case of 26 OECD countries for the period 2000–2012. Muldoon et al. [27] found that higher physician density rate worked as the significant reducing factor of infant and child mortality rate in the context of 136 UN member countries, where a mixed effects linear regression model based on 2008 cross-sectional data is employed. In the same way, Farahani et al. [25] found that the physician density reduces infant mortality both in the short run and long run, where global data of 1960–2000 are used. Using semi-parametric analysis, Liebert and Mäder [26] also observed that higher physician density reduced infant mortality in Germany from the data of 1928–1936. Russo et al. [28] found that the supply of primary care physicians contributed to the reduction of infant mortality in Brazil during 2005–2012. Shetty and Shetty [29] also concluded that the number of doctors per capita had an opposing affiliation with the mortality rate in the case of Asian countries. The role of more physicians in the most industrialised countries to ensure lower mortality rate has not been observed in the past literature. Therefore, inclusion of the number of physicians or its density in death rate analysis is essential to reaffirm its role.

Access to clean water facilities can also influence the mortality rate (see [14, 16, 27, 30, 31], among others). Ezeh et al. [31] found that the risk of mortality from unimproved water was significantly higher in Nigeria, where pooled data of 2003, 2008 and 2013 of Nigeria Demographic and Health Survey were used. Likewise, applying ordinal logistic regression, Cheng et al. [30] observed that the access to water significantly reduced infant, child, and maternal mortality in the case of 193 countries. Similar results were also found by Muldoon et al. [27] for 136 UN member countries. Lu et al. [14] ascertained that improved water facilities reduced the infant mortality rate in the case of 84 developing economies during 1995–2013. In contrast, Shobande [16] found no significant impact of improved water sources on infant and child mortality in 23 African countries. More research related to the necessity of access to clean water in the panel of industrial countries is urgently needed, but is not found in the existing literature. These findings have emphasised the need for more exploration of the importance of access to clean water facilities on the mortality rate.

The importance of sanitation facilities on the mortality rate cannot be ignored and a number of studies have revealed their role (see [8, 14–16, 27, 30–32]; among others). Rahman et al. [15] found that sanitation facilities significantly reduced the crude death rate and infant mortality rate in the case of 15 SAARC-ASEAN countries for the data period of 1995–2014 where fixed effect, random effect and GMM estimator were employed. Ezeh et al. [31] also found the impact of unimproved sanitation facilities on mortality in Nigeria. Lu et al. [14] and Alemu [8] observed that improved sanitation facilities reduced infant mortality rate for 84 developing economies, and for 33 African countries, respectively. Likewise, Cavalcanti et al. [32] found that the household sanitary facilities reduced child mortality in Brazil. Furthermore, Shobande [16] discovered that sanitation facilities had a significant impact on infant and child mortality in 23 African countries. In the same way, Muldoon et al. [27] and Cheng et al. [30] found that access to sanitation facilities reduced infant, child, and maternal mortality for 136 UN member countries and for 193 countries, respectively. However, in the previous studies, the importance of sanitation facilities on death rate in terms of a panel of most industrialised countries has been ignored. Thus it is important to identify the nexus between sanitation facilities and the mortality rate as a way of implementing improved policy initiatives.

Education reduces the mortality rate by increasing consciousness and responsiveness, as explicated in a number of empirical works, for example Halpern-Manners et al. [10], Doniec et al. [34], Buckles et al. [33], Sajedinejad et al. [35], Ray and Linden (2020), Alemu [8], Farahani et al. [25], among others. Halpern-Manners et al. [10] observed the robust relationships between education and mortality in the case of the USA. Similarly, Doniec et al. [34] discerned that those in lower educational groups were significantly more likely to die in the case of 3 Eastern European countries covering the period of 1982–2013. Buckles et al. [33] also found that college education reduced the mortality in Vietnam. Furthermore, Sajedinejad et al. [35] identified that education reduced the maternal mortality rate in 179 countries Ray and Linden (2020) observed that the primary education rate significantly reduced infant mortality in 195 countries during the data period of 1995–2014. Similar statistics were also been obtained by Alemu [8] in the case of 33 African countries from 1994 to 2013. However, Farahani et al. [25] and Anwar et al. [18] found no statistically significant impact of education on infant and neonatal mortality for the global panel and the 12 most vulnerable Asian countries, respectively. By critically analysing the research, it has been observed that past studies did not consider the significance of education in determining the death rate in the panel of most industrialised countries. Therefore, because the nexus between education and mortality is not clear, there is a need for investigations into this issue.

Urbanization can also play a significant role in the mortality rate. In this perspective, Bandyopadhyay and Green [9] found evidence of robust negative correlation between crude death rate and urbanization in the case of the global context. However, Brueckner [36] found no significant negative correlation between adult mortality and urbanization in sub-Saharan Africa during the data period of 1960–2013. Similarly, Wang [37] found that the increase in urbanization is significantly linked with reduced mortality, under-five mortality, and infant mortality in 163 countries during 1990–2012, and the effect is stronger in high-income nations. In contrast, Cavalcanti et al. [32] observed that the urbanization rate significantly increases child mortality in Brazil. Similarly, Taghizadeh-Hesary et al. [17] found that the urbanization rate increased the risks of lung and respiratory diseases in the case of 18 Asian countries. However, Shobande [16], and Muldoon et al. [27] found no significant impact of urbanization on infant and child mortality for 23 African countries, and for 136 UN member countries, respectively. The consideration of urbanization in the context of the panel of highly industrial countries has not been found in previous literature. Moreover, the ambiguous identification of impact of urbanization on mortality rate demands more thorough investigation.

From the current literature it may be observed that the existing identifications regarding the impact of industrial pollution, density of physicians, access to clean water and sanitation facilities, education, and the urbanization rate on human health status are not unanimous and conclusive. Furthermore, the findings of the noted determining factors on the death rate as a group in the world’s most 20 industrialised countries are absent. Therefore, the present study is an endeavour to fill up the existing literature gap to formulate efficacious and durable policy in the health sector.

## Data and methodologies

### Selection of countries

This study explores the relationship between health status and industrial pollution in the world’s 20 most industrialised countries. Based on manufacturing, value added (current US$), these industrialised countries^1^ are selected. The countries are China, United States, Japan, Germany, South Korea, India, Italy, France, United Kingdom, Mexico, Indonesia, Russia, Brazil, Canada, Spain, Turkey, Thailand, Switzerland, Ireland and Netherland.

### Unbalanced panel data

This is a panel data study covering the data period 1960–2019. Our data are unbalanced due to the unavailability of all data for the entire period for all sample countries. The data for this study are collected from the World Development Indicator [6] published by the World Bank. The used data for the selected variables are on crude death rate (per 1000 people), CO2 emissions from manufacturing industries and construction (% of total fuel combustion), nitrous oxide emissions (thousand metric tons of CO2 equivalent), gross domestic product (GDP) per capita (constant 2010 US$), physicians (per 1000 people), urban population (% of total population), people using at least basic drinking water services (% of population), people using at least basic sanitation services (% of population) and secondary school enrolment (% of gross).

### Theory, data, econometric approach and estimation software

Grossman [55] introduces the health production function, which explains the link between health input and an individual’s health output. The individual health production function can be explained as below:

1 $$ \mathrm{HO}=f\left(\mathrm{HI}\right) $$

where HO indicates an individual’s health output and HI denotes input needed for an individual’s health. The above model investigates individual health outcome at the micro level. We examine the impact of industrial pollution on health status at the macro level. Following Majeed and Ozturk’s [56] study, we converted the above model to macro level. The health inputs are divided into economic, environmental and social factors ([56–58], which can be expressed by the below equation:

2 $$ \mathrm{HO}=f\left(\mathrm{Environmental},\mathrm{Economic},\mathrm{Social}\right) $$

Adding healthcare factors, we have extended the eq. (2). Hence, eq. (2) can be written as follows:

3 $$ \mathrm{HO}=f\left(\mathrm{Environmental},\mathrm{Economic},\mathrm{Healthcare},\mathrm{Social}\right) $$

We used two environmental variables that arose from industrial pollution: CO2 emissions from manufacturing industries and construction, and nitrous oxide emissions. We then developed two models using two industrial pollution variables. The first and second models consisted of CO2 emissions and Nitrous oxide emissions, respectively. In addition to environmental factors, we have also added a range of economic, healthcare and social factors in our models: GDP per capita (economic factor), doctor/population ratio, sanitation, drinking water (healthcare facility factors), secondary school enrolment and urbanisation (social factors). Hence, our models for empirical investigation is as follows:

4.1 $$ {\mathrm{DEA}}_{\mathrm{it}}={\beta}_1+{\beta}_2\mathrm{CO}{2}_{\mathrm{it}}+{\beta}_3{\mathrm{GDP}}_{\mathrm{it}}+{\beta}_4{\mathrm{PHY}}_{\mathrm{it}}+{\beta}_5{\mathrm{WAT}}_{\mathrm{it}}+{\beta}_6{\mathrm{SAN}}_{\mathrm{it}}+{\beta}_7{\mathrm{SCH}}_{\mathrm{it}}+{\beta}_8{\mathrm{URB}}_{\mathrm{it}}+{\varepsilon}_{\mathrm{it}} $$

4.2 $$ {\mathrm{DEA}}_{\mathrm{it}}={\beta}_1+{\beta}_2{\mathrm{NIT}}_{\mathrm{it}}+{\beta}_3{\mathrm{GDP}}_{\mathrm{it}}+{\beta}_4{\mathrm{PHY}}_{\mathrm{it}}+{\beta}_5{\mathrm{WAT}}_{\mathrm{it}}+{\beta}_6{\mathrm{SAN}}_{\mathrm{it}}+{\beta}_7{\mathrm{SCH}}_{\mathrm{it}}+{\beta}_8{\mathrm{URB}}_{\mathrm{it}}+{\varepsilon}_{\mathrm{it}} $$

where DEA is our dependent variable that represents the death rate. The right-hand side variables are explanatory variables where CO2, NIT, GDP, PHY, WAT, SAN, SCH and URB denote CO2 emissions, nitrous oxide emissions, GDP per capita, physicians, drinking water services, sanitation services, secondary school enrolment and urbanisation, respectively.

Following previous studies such as those of Majeed and Ozturk [56] and Siddique and Kiani [58] among others, we also took the logarithm of our variables. One of the main advantages of taking the logarithm is that coefficient estimates will provide the elasticities directly. Therefore, the models for our empirical study will be as follows:

5.1 $$ {\mathrm{lnDEA}}_{\mathrm{it}}={\beta}_1+{\beta}_2\mathrm{lnCO}{2}_{\mathrm{it}}+{\beta}_3{\mathrm{lnGDP}}_{\mathrm{it}}+{\beta}_4{\mathrm{lnPHY}}_{\mathrm{it}}+{\beta}_5{\mathrm{lnWAT}}_{\mathrm{it}}+{\beta}_6{\mathrm{lnSAN}}_{\mathrm{it}}+{\beta}_7{\mathrm{lnSCH}}_{\mathrm{it}}+{\beta}_4{\mathrm{lnURB}}_{\mathrm{it}}+{\varepsilon}_{\mathrm{it}} $$

5.2 $$ {\mathrm{lnDEA}}_{\mathrm{it}}={\beta}_1+{\beta}_2{\mathrm{lnNIT}}_{\mathrm{it}}+{\beta}_3{\mathrm{lnGDP}}_{\mathrm{it}}+{\beta}_4{\mathrm{lnPHY}}_{\mathrm{it}}+{\beta}_5{\mathrm{lnWAT}}_{\mathrm{it}}+{\beta}_6{\mathrm{lnSAN}}_{\mathrm{it}}+{\beta}_7{\mathrm{lnSCH}}_{\mathrm{it}}+{\beta}_4{\mathrm{lnURB}}_{\mathrm{it}}+{\varepsilon}_{\mathrm{it}} $$

Heteroscedasticity, serial correlations and cross-sectional dependences generally exist in panel data because of an increasing availability of data, rapid urbanization and industrialisation, improvement of sanitation and water facilities on a priority basis, better education opportunities, positive economic development, significant amount of industrial pollution, focus on public health issues and economic globalization. All these factors are more common in the case of world’s most industrialised countries. Moreover, if both the cross-section dimension *(N)* and the time series dimension (*T*) are large, countries’ economic development may be mutually dependent. Therefore, ignoring the heteroscedasticity, the serial correlations and the cross-sectional dependences may provide inefficient statistical inference [59].

The standard fixed effect model will not be able to produce robust results if a panel data set contains heteroscedasticity, autocorrelation, and cross-sectional dependence. Therefore, this study adopts Hoechle’s [60] procedure of the STATA xtscc program that produces Driscoll and Kraay’s [38] standard error technique for linear panel models. These are consistent for heteroskedasticity and also robust to general forms of cross-sectional dependence to examine the impact of industrial pollution on health status for a panel of industrial countries. For applying Driscoll and Kraay’s [38] standard error technique, this study follows a two-steps procedure. The average values obtained from the product of independent variables and residuals is the first step. These values in a weighted heteroskedasticity autocorrelation (HAC) estimator will be used to generate standard errors, which now have the added feature of being robust against cross-sectional dependence in the second step [61–63]. There are a number of advantages of having Driscoll and Kraay’s [38] standard error technique. First, it is one of the best techniques if there is any scope of heteroscedasticity, cross-sectional dependency and serial correlation in the data [64–66]. Second, this technique is a non-parametric approach which accommodates flexibility, and large time dimension. Third, this technique can apply in both balanced and unbalanced panel data. Finally, this technique can handle missing values [60].

Following the methodology of Le and Nguyen [67]; Ikpesu et al. [68] we have also employed the Panel-corrected standard error (PCSE) technique for robustness checking and validating our outcomes.

A series of econometric procedures have been applied to check the unbalanced panel data. First, the study checks the presence of heteroscedasticity, serial correlation, and cross-sectional dependence in the panel data. Modified Wald statistics for groupwise heteroskedasticity will be used to see the existence of heteroscedasticity in the data set [69]. The presence of serial correlation will be tested using Wooldridge [70]. Pesaran [71] CD statistic is a diagnostic test that checks the existence of cross-sectional dependence. Only Pesaran’s CD test is adequate while using unbalanced panels [60]. Pesaran’s [71] CD test is given as below:

6 $$ CD=\sqrt{\frac{2T}{N\left(N-1\right)}}\ \left({\sum}_{i=1}^{N-1}{\sum}_{j=i+1}^N\sqrt{T_{ij}{\hat{p_{ij}}}^2}\right) $$

Where ${\hat{p_{ij}}}^2$ represents the pairwise cross-sectional correlation coefficient of residuals, and *T* and *N* represent the time and cross-sectional dimensions of the panel, respectively. In this setting, the null hypothesis has cross-sectional independence with CD ^~^ *N* (0, 1).

## Empirical results

### Descriptive statistics

Table 1 presents the descriptive statistics of the variables. The average (median) value of crude death rate is 9.05 (8.70). The minimum and maximum of crude death rate are 4.69 and 25.43, respectively. The mean and median values of CO2 emissions are 21.80 and 21.16, respectively. The average (median) value of nitrous oxide emissions is 83.65 (38.09). The average of GDP per capita, physicians, urbanisation, drinking water services, sanitation services and school enrolment are 21,267.66, 1.99, 62.42, 96.43, 89.11 and 82.71, respectively.

**Table 1:** Descriptive Statistics

Variable	Mean	Median	Std. Dev.	Minimum	Maximum	Observation
DEA	9.05	8.70	2.69	4.69	25.43	1180
CO2	21.80	21.16	7.81	7.96	49.15	993
NIT	83.65	38.09	103.78	2.39	587.17	860
GDP	21,267.66	17,442.06	18,365.67	132.07	79,703.41	1131
PHY	1.99	1.81	1.17	0.02	6.63	741
URB	62.42	71.10	19.63	14.59	91.88	1200
WAT	96.43	98.88	5.41	75.65	100.00	353
SAN	89.11	98.04	17.07	16.37	100.00	354
SCH	82.71	91.47	27.28	18.13	140.69	745

### Results of heteroscedasticity and autocorrelation

Table 2 presents the results of heteroscedasticity and autocorrelation. The presences of heteroscedasticity, serial correlation and cross-sectional dependence in panel data have serious problems for econometric analysis. Heteroscedasticity exists when the variance of the disturbance differs across samples [72]. Autocorrelation is the error term correlated with any variable of the model which is not affected by the error term related to other variables in this model [73]. Table 2 ensures the existence of heteroscedasticity and autocorrelation.

**Table 2:** Results of diagnostic tests for heteroscedasticity and autocorrelation

	Test statistic	
Test	CO2 emissions equation (5.1)	Nitrous oxide emissions eq. (5.1)
Modified Wald test for groupwise heteroskedasticity	425.29^****^	3355.33^***^
Wooldridge test for autocorrelation in panel data	5.538^**^	4.705^**^

Heteroscedasticity: Modified Wald test for groupwise heteroskedasticity; Ho: sigma(i)^^^ 2 = sigma^^^ 2 for all i: No heteroskedasticity. Auto correlation: Wooldridge test for autocorrelation in panel data. H0: no first-order autocorrelation. Note: ^***^ and ^**^ denote significance level at 1 and 5%, respectively

### Results of cross-sectional dependence test

Table 3 reports Pesaran’s [71] cross-sectional dependence test results. The presence of cross-sectional dependence in a panel study suggests the existence of common unobserved shock among the cross-sectional variables over a time period [74]. The results show that the null hypothesis of cross-sectional dependence is rejected at the 1% statistical significance level for all variables used in this study implying that there is strong evidence of the presence of cross-sectional dependence.

**Table 3:** Pesaran [71] CD test for cross-sectional dependence

Variables	CD test	*p*-value
DEA	27.876^***^	0.000
CO2	49.870^***^	0.000
NIT	18.372^***^	0.000
GDP	93.776^***^	0.000
PHY	39.419^***^	0.000
URB	91.624^***^	0.000
WAT	19.473^***^	0.000
SAN	4.234^***^	0.000
SCH	39.806^***^	0.000

Notes: Under the null hypothesis of cross-section independence, CD ~ N(0,1) *P*-values close to zero indicate data are correlated across panel groups. ^***^ indicates rejection of the null hypothesis at the 1% significance level

### Results of Driscoll-Kraay standard error estimation

This study estimates regression results using Hoechle’s [60] procedure with Driscoll-Kraay’s [38] robust standard error to validate the statistical inferences. All variables except GDP per capita and urbanization are found to be significant at the 1% level while using Eq. (5.1) in Model 1 of Table 4. The GDP per capita and urbanization are significant at the 5% level. Carbon emissions positively and significantly contribute to industrial pollution which leads to an increase in the crude death rate, suggesting that a 1% increase in CO2 emissions increases crude death rate by 0.10% in the world’s twenty most industrialised countries. This result is comparable with that of Siddique and Kiani [58] who found that a 1% increases in CO2 emissions increases infant mortality rate by 0.14, 0.09 and 0.26% in middle-income, upper-middle-income and lower-middle-income countries, respectively.

**Table 4:** Estimation results: Driscoll-Kraay [38] standard errors

Models	1	2
Constant	−8.8219 (−5.86)^***^	−6.6460 (− 6.57)^***^
CO2	0.0988 (5.67)^***^	–
NIT	–	0.0687 (4.13)^***^
GDP	− 0.0648 (−2.56)^**^	−0.0757 (− 3.80)^***^
PHY	− 0.1214 (−5.41)^***^	− 0.1302 (− 7.12)^***^
URB	− 0.1311 (− 2.00)^**^	− 0.1723 (− 2.30)^**^
WAT	3.1370 (7.06)^***^	2.4197 (7.68)^***^
SAN	− 0.4528 (−6.41)^***^	− 0.3938 (− 6.28)^***^
SCH	− 0.1003 (− 3.58)^***^	0.0473 (1.46)
within R-squared	0.4945	0.4550
F-statistic	606.57	414.64
Probability	0.0000	0.0000
Number of groups	19^a^	19
Number of observations	225	197

Note: ^***^ and ^**^ denote significance level at 1 and 5%, respectively

^a^Japan does not have the secondary school enrolment data for the studied period, therefore, the statistical software and estimation techniques used in this study exclude Japan from this analysis.

The impacts of a number of factors on crude death rate: economic growth, availability of physicians, urbanization, accessible sanitation and education are negative and statistically significant indicating that 1% increase in these factors decreases crude death rate by 0.07, 0.12, 0.13, 0.45 and 0.10%, respectively. Since the average income of workers in industrial countries is high, they are likely to have better resources and technologies to reduce the death rate arising from industrial pollution. The residents from industrialised countries can have more accessibility to doctors and healthcare facilities that reduce the death rate from industrial pollutions. Urbanization, sanitation and education have positive effects on health status [58] that mitigate the crude death rate. Our obtained results confirm this proposition empirically.

Model 2 of Table 4 shows the results using Eq. (5.2). The effect of nitrous oxide emissions on crude death rate is positive and statistically significant at the 1% level implying that a 1% increase in of nitrous oxide emissions increases crude death rate by 0.07% in the sample countries. This result is also comparable with that of Siddique and Kiani [58] who find that a 1% increase in nitrous oxide emissions increases infant mortality rate by 0.21, 0.24 and 0.19% in middle-income, upper-middle-income and lower-middle-income countries, respectively. Economic growth, access to physicians, urbanization and sanitation have a negative and significant effect on the crude death rate suggesting that 1% increase in these areas decreases the crude death rate by 0.08, 0.13, 0.17 and 0.39%, respectively. Our findings of economic growth and sanitation facilities are in line with those of Rahman et al. [15] and Kengnal and Holyachi [75], and the effect of access to physicians is consistent with the findings of Shahid et al. [76]. However, our result in relation to urbanisation is contradictory to the findings of Li et al. [77]. Surprisingly, we have obtained a positive association between crude death rate and basic drinking water services. This result is unexpected and contradictory to the findings of Majeed and Ozturk [56] who investigated the relationship between infant mortality and safe drinking water, using a global panel sample for the period 1990–2016, and found a negative relationship between infant mortality rate and safe drinking water.

### Robustness check

Panel-corrected standard error (PCSE) technique effectively deals with heteroscedasticity, serial correlations, and cross-sectional dependence [67, 68]. Therefore, we use PCSE estimation as robustness test to compare our results. Table 5 reports the results of PCSE.

**Table 5:** The results of PCSE regression

Models	1	2
Constant	−10.7364 (−9.50^***^	−8.1999 (−6.66)^***^
CO_2_	0.0945 (5.92)^***^	
NIT	–	0.0637 (4.61)^***^
GDP	−0.1251 (−3.86)^***^	− 0.1112 (− 3.41)^***^
PHY	− 0.1072 (−4.74)^***^	−0.1052 (−6.11)^***^
URB	−0.0828 (−1.19)	−0.0943 (− 1.45)
WAT	3.6369 (11.53)^***^	2.8050 (8.13)^***^
SAN	−0.4754 (−12.12)^***^	− 0.3948 (− 10.58)^***^
SCH	− 0.0733 (− 1.68)^*^	0.0155 (0.32)
R-squared	0.9820	0.9830
F-statistic	533.69	585.08
Probability	0.0000	0.0000
Number of groups	19	19
Number of observations	225	197

Note: ***, **, and * denote significance level at 1%, 5% and 10%, respectively

Carbon emissions, nitrous oxide emissions and water have significant positive effects on crude death, which are consistent with the results of Table 4. The economic growth, access to physicians, and sanitation have a negative and significant effect on the crude death rate. Overall, the results from the PCSE estimate show consistent results with the results of Driscoll-Kraay’s [38] robust standard error estimates.

## Conclusions and policy implications

The current study explores the link between health status and industrial pollution in the world’s 20 most industrialised countries, controlling for some other variables. Crude death rate is used to represent health status, and CO_2_ emissions from manufacturing industries and construction, and nitrous oxide emissions are considered as markers for industrial pollution. Using annual data for 60 years (1960–2019), an unbalanced panel data estimation method is followed where Driscoll and Kraay’s [38] standard error technique is employed to deal with heteroscedasticity, autocorrelation and cross-sectional dependence problems. The research findings indicate that industrial pollution arising from both variables significantly increases the death rate, while an increase in economic growth, number of physicians, urbanisation, sanitation facilities and access to schooling decreases the death rate. Therefore, minimisation of industrial pollution should be the topmost policy agenda in these countries. All the findings are consistent theoretically, and have empirical implications as well. These outcomes also comply with the policy guidelines of United Nations Development Program (UNDP) and World Health Organization (WHO) in addressing industrial pollution along with other factors to substantially reduce the death rate by 2030 and improve the public health status (SDGs’ target 3.9 of Goal 3, [78, 79]). The policy implication of this study is: the mitigation of industrial pollution, considering other pertinent factors, should be addressed appropriately by enunciating effective policies to reduce the human death rate and improve health status in the studied panel countries. The following particular recommendations will be useful in this respect:

i. *Reducing industrial pollution:* The reduction of industrial pollution (CO_2_ and nitrous oxide emissions) is essential in relation to environmentally friendly initiatives, which can play a role in reducing pollution related diseases, and eventually diminish the death rate. In this regard, effective pollution disposal facilities should be introduced and technology should be developed to convert industrial wastages into fresh materials and polluted smoke into clean air. A complete and wide-ranging policy package on reducing industrial pollution should be formulated and executed.
ii. *Sustainable economic development:* Sustainable economic development along with environmental considerations is required to make the earth habitable for future generations. In this perspective, green development, green growth, green technology and a pollution free environment are urgently needed to improve the habitat for humans and thus reduce the mortality rate. Thus efficient and inclusive policy initiatives to ensure sustainable development will be essential for improving the health status of people by reducing the death rate.
iii. *Increasing physician numbers:* Physicians provide better medical services and guidelines for taking proper drugs and medicines leading to cures from various diseases and improving living standards, which both decrease the mortality rate. In this perspective, a concrete policy venture for generating abundant physician numbers and ease access to them to protect human health is essential.
iv. *Enhancing access to clean water and sanitation facilities:* Wide access to clean water and better sanitation facilities is required to decrease the mortality rate, as contaminated drinking water and unhygienic sanitation may cause an increase in the death rate. A well-thought out and well-accepted policy initiative is required to facilitate access to clean water and sanitation facilities to all people.
v. *Enlarging educational opportunities:* Educational facilities increase awareness of medical and health consciousness to protect people from various fatal diseases and therefore reduce the death rate. An effective policy formulation is necessary to enlarge widespread educational facilities among the entire population to reduce the mortality rate.
vi. *Well-organized urbanization:* Well-planned urban facilities may increase living standards and increase quality of life by establishing well-organized housing, industries, hospitals, supplying electricity, clean water and hygienic sanitation facilities. Therefore, a complete well-organized urbanization policy design is urgently needed in conjunction with other policies.

## Data Availability

**The datasets used and/or analysed during the current study are available from the corresponding author on reasonable request.**

## Footnotes

1 Data source for these countries: World Bank national accounts data, and OECD National Accounts data files https://www.indexmundi.com/facts/indicators/NV.IND.MANF.CD/rankings

## References

[1] Gillespie, C. (2019). Examples of Secondary Pollutants. Sciencing. https://sciencing.com/examples-secondary-pollutants-5314906.html

[2] Kelishadi R (2012). Environmental pollution: health effects and operational implications for pollutants removal. J Environ Public Health.

[3] UNDP (2020). Goal 3: good health and well-being.

[4] Mudu P, Terracini B, Martuzzi M (2014). Human health in areas with industrial contamination.

[5] Ukaogo PO, Ewuzie U, Onwuka CV. Environmental pollution: causes, effects, and the remedies. Microorganisms for Sustainable Environment and Health. 2020:419–29. 10.1016/b978-0-12-819001-2.00021-8.

[6] WDI (2020). World development indicators.

[7] BP (2020). Statistical Review of World Energy: Carbon dioxide emissions. https://www.bp.com/content/dam/bp/business-sites/en/global/corporate/pdfs/energy-economics/statistical-review/bp-stats-review-2020-co2-emissions.pdf

[8] Alemu AM (2017). To what extent does access to improved sanitation explain the observed differences in infant mortality in Africa?. Afr J Primary Health Care Family Med.

[9] Bandyopadhyay S, Green E (2018). Urbanization and mortality decline. J Reg Sci.

[10] Halpern-Manners A, Helgertz J, Warren JR, Roberts E (2020). The effects of education on mortality: evidence from linked U.S. Census and administrative mortality data. Demography.

[11] Jebeli SSH, Hadian M, Souresrafil A (2019). Study of health resource and health outcomes: organization of economic corporation and development panel data analysis. J Education Health Promotion.

[12] Karimi B, Shokrinezhad B (2020). Air pollution and mortality among infant and children under five years: a systematic review and meta-analysis. Atmospheric Pollution Res.

[13] Leogrande S, Alessandrini ER, Stafoggia M, Morabito A, Nocioni A, Ancona C, Bisceglia L, Mataloni F, Giua R, Mincuzzi A, Minerba S, Spagnolo S, Pastore T, Tanzarella A, Assennato G, Forastiere F (2019). Industrial air pollution and mortality in the Taranto area, southern Italy: a difference-in-differences approach. Environ Int.

[14] Lu Z, Bandara JS, Paramati SR (2019). Impact of sanitation, safe drinking water and health expenditure on infant mortality rate in developing economies. Aust Econ Pap.

[15] Rahman MM, Khanam R, Rahman M (2018). Health care expenditure and health outcome nexus: new evidence from the SAARC-ASEAN region. Glob Health.

[16] Shobande OA (2020). The effects of energy use on infant mortality rates in Africa. Environmental Sustainability Indicators.

[17] Taghizadeh-Hesary F, Rasoulinezhad E, Yoshino N, Chang Y, Taghizadeh-Hesary F, Morgan PJ. The energy–pollution–health nexus: A panel data analysis of low- and middle-income Asian countries. Singap Econ Rev. 2020;66(02):435–55. 10.1142/s0217590820430043.

[18] Anwar A, Ayub M, Khan N, Flahault A (2019). Nexus between air pollution and neonatal deaths: a case of Asian countries. Int J Environ Res Public Health.

[19] Bauleo L, Bucci S, Antonucci C, Sozzi R, Davoli M, Forastiere F, Ancona C (2018). Long-term exposure to air pollutants from multiple sources and mortality in an industrial area: a cohort study. Occup Environ Med.

[20] Lehtomäki H, Geels C, Brandt J, Rao S, Yaramenka K, Åström S, Andersen MS, Frohn LM, Im U, Hänninen O (2020). Deaths attributable to air pollution in Nordic countries: disparities in the estimates. Atmosphere.

[21] Næss Ø, Nafstad P, Aamodt G, Claussen B, Rosland P (2006). Relation between concentration of air pollution and cause-specific mortality: four-year exposures to nitrogen dioxide and particulate matter pollutants in 470 neighborhoods in Oslo, Norway. Am J Epidemiol.

[22] Rajak R, Chattopadhyay A (2019). Short and long term exposure to ambient air pollution and impact on health in India: a systematic review. Int J Environ Health Res.

[23] Shan A, Zhang Y, Zhang L, Chen X, Li X, Wu H, Yan M, Li Y, Xian P, Ma Z, Li C, Guo P, Dong G, Liu Y, Chen J, Wang T, Zhao B, Tang N (2020). Associations between the incidence and mortality rates of type 2 diabetes mellitus and long-term exposure to ambient air pollution: a 12-year cohort study in northern China. Environ Res.

[24] Fareed Z, Iqbal N, Shahzad F, Shah SGM, Zulfiqar B, Shahzad K, Hashmi SH, Shahzad U (2020). Co-variance nexus between COVID-19 mortality, humidity, and air quality index in Wuhan, China: new insights from partial and multiple wavelet coherence. Air Quality, Atmosphere & Health.

[25] Farahani M, Subramanian SV, Canning D. Short and long-term relationship between physician density on infant mortality: a longitudinal econometric analysis. PGDA working paper no. 49. National Institute on Aging. 2009. https://cdn1.sph.harvard.edu/wp-content/uploads/sites/1288/2013/10/PGDA_WP_49.pdf.

[26] Liebert, H. & Mäder, B. (2018). Physician density and infant mortality: a semiparametric analysis of the returns to health care provision. CESifo working paper no. 7209, available at SSRN: https://ssrn.com/abstract=3275382

[27] Muldoon KA, Galway LP, Nakajima M, Kanters S, Hogg RS, Bendavid E, Mills EJ (2011). Health system determinants of infant, child and maternal mortality: a cross-sectional study of UN member countries. Glob Health.

[28] Russo LX, Scott A, Sivey P, Dias J (2019). Primary care physicians and infant mortality: evidence from Brazil. PLoS One.

[29] Shetty A, Shetty S (2014). The impact of doctors per capita on the mortality rate in Asia. Int J Med Pharmaceutical Sci.

[30] Cheng JJ, Schuster-Wallace CJ, Watt S, Newbold BK, Mente A. An ecological quantification of the relationships between water, sanitation and infant, child, and maternal mortality. Environ Health. 2012;11(4). 10.1186/1476-069x-11-4. 10.1186/1476-069X-11-4 PMC3293047 22280473

[31] Ezeh O, Agho K, Dibley M, Hall J, Page A (2014). The impact of water and sanitation on childhood mortality in Nigeria: evidence from demographic and health surveys, 2003–2013. Int J Environ Res Public Health.

[32] Cavalcanti A, Teixeira A, Pontes K (2019). Regression model to evaluate the impact of basic sanitation Services in Households and Schools on child mortality in the municipalities of the state of Alagoas, Brazil. Sustainability.

[33] Buckles K, Hagemann A, Malamud O, Morrill M, Wozniak A (2016). The effect of college education on mortality. J Health Econ.

[34] Doniec K, Stefler D, Murphy M, Gugushvili A, McKee M, Marmot M, Bobak M, King L (2018). Education and mortality in three eastern European populations: findings from the PrivMort retrospective cohort study. Eur J Pub Health.

[35] Sajedinejad S, Majdzadeh R, Vedadhir AA, Tabatabaei M, Mohammad K (2015). Maternal mortality: a cross-sectional study in global health. Glob Health.

[36] Brueckner M (2019). Adult mortality and urbanization: examination of a weak connection in sub-Saharan Africa. World Dev.

[37] Wang Q (2018). Urbanization and Global Health: the role of air pollution. Iran J Public Health.

[38] Driscoll JC, Kraay AC (1998). Consistent covariance matrix estimation with spatially dependent panel data. Rev Econ Stat.

[39] Brito J, Bernardo A, Zagalo C, Gonçalves LL (2021). Quantitative analysis of air pollution and mortality in Portugal: current trends and links following proposed biological pathways. Sci Total Environ.

[40] Clancy L, Goodman P, Sinclair H, Dockery DW (2002). Effect of air-pollution control on death rates in Dublin, Ireland: an intervention study. Lancet.

[41] Duong HH, Jayanthakumaran K (2018). Output, environmental pollution and health nexus in Vietnam: an estimation of simultaneous model with panel data. Int J Bonds Derivatives.

[42] Dedoussi IC, Eastham SD, Monier E, Barrett SRH (2020). Premature mortality related to United States cross-state air pollution. Nature.

[43] Im U, Christensen JH, Nielsen O-K, Sand M, Makkonen R, Geels C, Anderson C, Kukkonen J, Lopez-Aparicio S, Brandt J (2019). Contributions of Nordic anthropogenic emissions on air pollution and premature mortality over the Nordic region and the Arctic. Atmos Chem Phys.

[44] Coker ES, Cavalli L, Fabrizi E, Guastella G, Lippo E, Parisi ML, Pontarollo N, Rizzati M, Varacca A, Vergalli S (2020). The effects of air pollution on COVID-19 related mortality in northern Italy. Environ Resour Econ.

[45] Isphording, I. E. & Pestel, N. (2020). Pandemic meets pollution: poor air quality increases deaths by COVID-19. CESifo working paper no. 8495, available at SSRN: https://ssrn.com/abstract=3680352 10.1016/j.jeem.2021.102448 PMC8028850 33850337

[46] Marquès M, Rovira J, Nadal M, Domingo JL (2021). Effects of air pollution on the potential transmission and mortality of COVID-19: a preliminary case-study in Tarragona Province (Catalonia, Spain). Environ Res.

[47] Gupta A, Bherwani H, Gautam S, Anjum S, Musugu K, Kumar N, Anshul A, Kumar R (2020). Air pollution aggravating COVID-19 lethality? Exploration in Asian cities using statistical models. Environ Dev Sustain.

[48] Karuppasamy MB, Seshachalam S, Natesan U, Ayyamperumal R, Karuppannan S, Gopalakrishnan G, Nazir N (2020). Air pollution improvement and mortality rate during COVID-19 pandemic in India: global intersectional study. Air Quality Atmosphere & Health.

[49] Cheung CW, He G, Pan Y (2020). Mitigating the air pollution effect? The remarkable decline in the pollution-mortality relationship in Hong Kong. J Environ Econ Manag.

[50] Mehmood U (2020). Globalization-driven CO2 emissions in Singapore: an application of ARDL approach. Environ Sci Pollut Res.

[51] Mehmood U, Tariq S (2020). Globalization and CO2 emissions nexus: evidence from the EKC hypothesis in south Asian countries. Environ Sci Pollut Res.

[52] Mehmood U, Tariq S, Ul-Haq Z, Meo MS (2020). Does the modifying role of institutional quality remains homogeneous in GDP-CO2 emission nexus? New evidence from ARDL approach. Environ Sci Pollut Res.

[53] Mehmood U (2021). Renewable-nonrenewable energy: institutional quality and environment nexus in south Asian countries. Environmental Science and Pollution Research Published.

[54] Mehmood U, Mansoor A, Tariq S, Ul-Haq Z (2021). The interactional role of globalization in tourism-CO2 nexus in south Asian countries. Environmental Sci Pollution Res.

[55] Grossman M (1972). On the concept of health capital and the demand for health. J Polit Econ.

[56] Majeed MT, Ozturk I. Environmental degradation and population health outcomes: a global panel data analysis. Environ Sci Pollut Res. 2020;27(13):15901–15911. 10.1007/s11356-020-08167-8. 10.1007/s11356-020-08167-8 32100215

[57] Fayissa B, Gutema P (2005). Estimating a health production function for sub-Saharan Africa (SSA). Appl Econ.

[58] Siddique HMA, Kiani AK. Industrial pollution and human health: evidence from middle-income countries. Environ Sci Pollut Res. 2020:1–10. 10.1007/s11356-020-07657-z 31997247

[59] Qiu J, Ma Q, Wu L (2019). A moving blocks empirical likelihood method for panel linear fixed effects models with serial correlations and cross-sectional dependences. Econ Model.

[60] Hoechle D (2007). Robust standard errors for panel regressions with cross-sectional dependence. Stata J.

[61] Baloch MA, Meng F, Zhang J, Xu Z (2018). Financial instability and CO_2_ emissions: the case of Saudi Arabia. Environ Sci Pollut Res.

[62] Jalil A (2014). Energy–growth conundrum in energy exporting and importing countries: evidence from heterogeneous panel methods robust to cross-sectional dependence. Energy Econ.

[63] Özokcu S, Özdemir Ö (2017). Economic growth, energy, and environmental Kuznets curve. Renew Sust Energ Rev.

[64] Baloch MA, Meng F (2019). Modeling the non-linear relationship between financial development and energy consumption: statistical experience from OECD countries. Environ Sci Pollut Res.

[65] Baloch MA, Zhang J, Iqbal K, Iqbal Z (2019). The effect of financial development on ecological footprint in BRI countries: evidence from panel data estimation. Environ Sci Pollut Res.

[66] Sarkodie SA, Strezov V (2019). Effect of foreign direct investments, economic development and energy consumption on greenhouse gas emissions in developing countries. Sci Total Environ.

[67] Le T-H, Nguyen CP (2019). Is energy security a driver for economic growth? Evidence from a global sample. Energy Pol.

[68] Ikpesu, F, Vincent, O & Dakare, O 2019, 'Growth effect of trade and investment in Sub-Saharan Africa countries: Empirical insight from panel corrected standard error (PCSE) technique', Cogent Economics & Finance, vol. 7, no. 1, p. 1607127.

[69] Baum CF (2001). Residual diagnostics for cross-section time series regression models. Stata J.

[70] Wooldridge JM. Econometric analysis of cross section and panel data, vol. 108. Cambridge: MIT press; 2002.

[71] Pesaran MH (2004). General diagnostic tests for cross section dependence in panels.

[72] Simpson D (2012). Knowledge resources as a mediator of the relationship between recycling pressures and environmental performance. J Clean Prod.

[73] Attari MIJ, Hussain M, Javid AY (2016). Carbon emissions and industrial growth: an ARDL analysis for Pakistan. Int J Energy Sector Management.

[74] Akadiri, S. S. & Akadiri, A. C. (2019). Examining the causal relationship between tourism, exchange rate, and economic growth in tourism island states: evidence from second-generation panel. International Journal of Hospitality & Tourism Administration**,** 1-16.

[75] Kengnal P, Holyachi S (2017). The causal relationship between infant mortality rate, health expenditure and economic growth in India. Int J Public Health Res.

[76] Shahid A, Siddique HMA, Liaqat R (2019). Human health and foreign direct investment Nexus: evidence from South Asia. Asian Development Pol Rev.

[77] Li X, Wang C, Zhang G, Xiao L, Dixon J (2012). Urbanisation and human health in China: spatial features and a systemic perspective. Environ Sci Pollut Res.

[78] UNDP. (2015). Sustainable Development Goals. https://sustainabledevelopment.un.org/content/documents/21252030%20Agenda%20for%20Sustainable%20Development%20web.pdf

[79] WHO. (2016). Sustainable development goals (SDGs) : Goal 3. Target 3.9: By 2030, Substantially reduce the number of deaths and illnesses from hazardous chemicals and air, water and soil pollution and contamination [poster]. World Health Organization. https://iris.wpro.who.int/handle/10665.1/12881

